# Clinical and biomarker analyses of SHR-1701 combined with famitinib in patients with previously treated advanced biliary tract cancer or pancreatic ductal adenocarcinoma: a phase II trial

**DOI:** 10.1038/s41392-024-02052-3

**Published:** 2024-12-13

**Authors:** Lixia Yi, Haoqi Pan, Zhouyu Ning, Litao Xu, Hena Zhang, Longfei Peng, Yaowu Liu, Yifan Yang, Waimei Si, Ying Wang, Xiaoyan Zhu, Shenglin Huang, Zhiqiang Meng, Jing Xie

**Affiliations:** 1 Department of Integrative Oncology, Fudan University Shanghai Cancer Center, Shanghai, China; 2 Department of Minimally Invasive Therapy Center, Fudan University Shanghai Cancer Center, Shanghai, China; 3 Department of Oncology, Shanghai Medical College, Fudan University, Shanghai, China; 4 Shanghai Key Laboratory of Medical Epigenetics, Institutes of Biomedical Sciences, Fudan University, Shanghai, China; 5 Suzhou Institute of Biomedical Engineering and Technology, Chinese Academy of Sciences, Suzhou, China; 6 Clinical Research & Development, Jiangsu Hengrui Pharmaceuticals Co., Ltd., Shanghai, China

**Keywords:** Gastrointestinal cancer, Drug development

## Abstract

Advanced biliary tract cancer (BTC) and pancreatic ductal adenocarcinoma (PDAC) have poor prognoses and limited treatment options. Here, we conducted this first-in-class phase II study to evaluate the efficacy and safety of SHR-1701, a bifunctional fusion protein targeting programmed death-ligand 1 (PD-L1) and transforming growth factor-beta (TGF-β), combined with famitinib, a multi-targeted receptor tyrosine kinase inhibitor, in patients with advanced BTC or PDAC who failed previous standard treatment (trial registration: ChiCTR2000037927). Among 51 enrolled patients, the BTC cohort showed an objective response rate (ORR) of 28% (including 2 complete responses) and a disease control rate (DCR) of 80%, with a median progression-free survival (mPFS) of 5.1 months and a median overall survival (mOS) of 16.0 months. In the PDAC cohort, the ORR was 15% (2 complete responses), with a DCR of 60%, and the mPFS and mOS were 2.1 months and 5.3 months, respectively. Grade 3 or 4 treatment-related adverse events (TRAEs) occurred in 29.4% of patients, with no grade 5 TRAEs reported. Exploratory analyses revealed that primary tumor resection history, peripheral blood immunophenotype changes, and distinct immune-metabolic profiles were associated with treatment benefits. An immune/metabolism score integrating the features of six genes was developed as a predictive biomarker for immunotherapy response in multiple cohorts, allowing for the selection of patients most likely to experience positive outcomes from this therapy regimen. In conclusion, our study provides proof-of-concept data supporting the potential of SHR-1701 plus famitinib as an effective and safe subsequent-line therapy for refractory BTC and PDAC, highlighting the promise of targeting PD-L1, TGF-β, and angiogenesis pathways simultaneously.

## Introduction

Biliary tract cancer (BTC), which includes cholangiocarcinoma, gallbladder cancer, and ampullary cancer, accounts for approximately 0.6% of all new cancer diagnoses in 2023, while the more common pancreatic ductal adenocarcinoma (PDAC) comprises nearly 3%.^1^ Both BTC and PDAC are aggressive malignancies with poor prognoses, often diagnosed at an advanced stage.^2–4^ Despite initial treatment with standard chemotherapy, disease progression is almost inevitable. For BTC, second-line treatment options include FOLFOX (folinic acid, fluorouracil, and oxaliplatin) chemotherapy following first-line cisplatin and gemcitabine, as well as targeted therapy for a subset of patients with specific genetic alterations.^2,3^ Advanced PDAC patients unresponsive to first-line treatment face constrained options, with nanoliposomal irinotecan in combination with fluorouracil and folinic acid (Nal-IRI with 5-FU/LV) as recommended.^4^ Current subsequent-line chemotherapies offer disappointing efficacy, with a median overall survival (mOS) of about 6.2 months for BTC and even lower for PDAC,^4,5^ and the modest survival benefit often comes at the cost of severe toxicities.

Although immunotherapy has revolutionized the treatment landscape for various solid tumors, immune checkpoint inhibitor (ICI) monotherapy has not yielded significant advantages in advanced BTC (objective response rates [ORR]: 3–20%) and PDAC ( ≤ 10%) due to the immunosuppressive tumor microenvironment (TME),^6,7^ with only specific subgroups being eligible.^8,9^ One of the key factors negatively shaping the TME is transforming growth factor-beta (TGF-β), a pleiotropic cytokine that promotes immune evasion by inhibiting cytotoxic T and NK lymphocytes, while inducing the differentiation of regulatory T cells (Tregs) and myeloid-derived suppressor cells.^10–12^ Elevated TGFB1 expression in tumor tissues has been linked to resistance to ICIs and poor prognosis.^13–15^

SHR-1701 is a novel bifunctional fusion protein composed of an anti-PD-L1 antibody fused with the extracellular domain of TGF-β receptor II, which functions as a “trap” for all three TGF-β isoforms. By simultaneously inhibiting both PD-L1 and TGF-β signalings, SHR-1701 has exhibited potential enhanced anti-tumor immunity in preliminary clinical studies of advanced solid tumors, such as cervical and gastric cancers, compared to PD1/PD-L1 monotherapies.^16–21^

Furthermore, angiogenesis is a known hallmark of cancer, making it an attractive intervention target for combination with immunotherapy. Famitinib, a multi-targeted tyrosine kinase inhibitor (TKI) of VEGFR2/3, PDGFR, and c-Kit, has shown promise in prolonging progression-free survival (PFS) when partnered with ICIs across various malignancies.^22^

Given the complementary mechanisms of SHR-1701 and famitinib, and the overlap profile between TGF-β activation, immune exhaustion, and VEGFA upregulation, we hypothesized that triple blockade of PD-L1, TGF-β and angiogenesis pathways might exert synergistic antitumor effects and improve clinical outcomes in patients.^23^ To date, no results have been reported for the regimen of bifunctional TGF-β/PD-L1 inhibitors with antiangiogenic drugs in solid tumors. This phase II trial aimed to evaluate the efficacy, safety, and potential predictive biomarkers of SHR-1701 plus famitinib in patients with refractory advanced BTC or PDAC who failed previous standard treatments.

## Results

### Patient characteristics

**Fig. 1:**
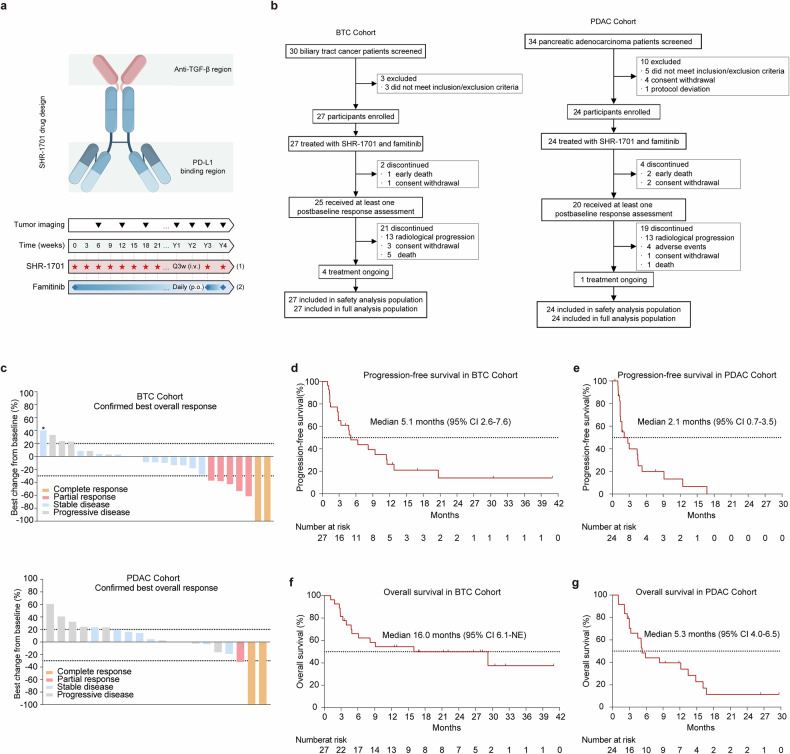
Study design and clinical outcomes of SHR-1701 plus famitinib in advanced BTC and PDAC. **a** Upper: Schematic representation of the bifunctional fusion protein SHR-1701. Lower: Treatment schedule of (1) SHR-1701 and (2) famitinib in the study. Q3w, every three weeks; i.v., intravenously; p.o., orally. **b** CONSORT (Consolidated Standards of Reporting Trials) diagram of patient disposition in the biliary tract cancer (BTC) and pancreatic ductal adenocarcinoma (PDAC) cohorts. Six patients were not evaluable for tumor response due to an early death (*n* = 3) or withdrawal of consent (*n* = 3) before the first scheduled post-baseline imaging assessment. **c** Waterfall plot of the best percent change from baseline in target lesion size and the best response for evaluable patients in the BTC and PDAC cohorts. *, tumor growth < 5 mm, still classified as stable disease per RECIST criteria. **d** Kaplan-Meier curve of progression-free survival (PFS) for the BTC cohort. Median PFS: 5.1 months (95% CI, 2.6–7.6). CI, confidence interval. **e** Kaplan-Meier curve of PFS for the PDAC cohort. Median PFS: 2.1 months (95% CI, 0.7-3.5). **f** Kaplan-Meier curve of overall survival (OS) for the BTC cohort. Median OS: 16.0 months (95% CI, 6.1-NE). NE, not estimable. **g** Kaplan-Meier curve of OS for the PDAC cohort. Median OS: 5.3 months (95% CI, 4.0–6.5)

Between September 2020 and April 2023, a total of 51 patients were enrolled, comprising 27 patients in the BTC cohort and 24 patients in the PDAC cohort, all of whom received the combination treatment of SHR-1701 and famitinib (Fig. 1a). At the data cutoff date of May 1, 2024, 45 patients (25 in the BTC cohort and 20 in the PDAC cohort) were assessable for response evaluation, while all 51 patients were included in the safety analysis (Fig. 1b). 2 patients in the BTC cohort and 4 patients in the PDAC cohort were excluded from the response assessment population due to early discontinuation of treatment before the first scheduled post-baseline imaging assessment. Treatment discontinuation occurred in 46 patients (90.2%), primarily due to disease progression. Advanced events (AE) led to treatment discontinuation in four patients (7.8%). Five patients were still receiving active treatment, with two patients continuing on both study drugs and three patients receiving famitinib monotherapy (Supplementary Fig. S1a and S1b).

Baseline characteristics were summarized in Table 1. In the overall population, the median age was 61 years, 52.9% of patients were male, and all patients had an Eastern Cooperative Oncology Group (ECOG) performance status of 1. In the BTC cohort, most patients had metastatic cancer in the liver (22/27, 81.5%), lungs (10/27, 37%), peritoneum (8/27, 29.6%), and distant lymph nodes (8/27, 29.6%). And the median number of prior systemic treatments at the start of therapy was 1. Fourteen patients (51.9%) had intrahepatic cholangiocarcinoma, 5 (18.5%) had extrahepatic cholangiocarcinoma, and 8 (29.6%) had gallbladder cancer. In the PDAC cohort, 75% (18/24) of patients experienced liver metastasis, 62.5% (15/24) experienced lung metastasis, and 54.2% (13/24) experienced peritoneal metastasis. And participants had received a median of two prior systemic treatments.

**Table 1:** Baseline demographic and clinical characteristics

	BTC cohort (*n* = 27)	PDAC cohort (*n* = 24)
**Age (years), median (range)**	62 (45–77)	61 (35–77)
**Gender,** ***n*** **(%)**		
Male	10 (37.0)	17 (70.8)
Female	17 (63.0)	7 (29.2)
**ECOG PS,** ***n*** **(%)**		
0	0	0
1	27 (100.0)	24 (100.0)
**Primary tumor histology,** ***n*** **(%)**		
Pancreatic ductal adenocarcinoma	–	24 (100.0)
Intrahepatic cholangiocarcinoma	14 (51.9)	–
Extrahepatic cholangiocarcinoma	5 (18.5)	–
Gallbladder adenocarcinoma	8 (29.6)	–
**Metastatic sites**		
Liver	22 (81.5)	18 (75.0)
Lung	10 (37.0)	15 (62.5)
Peritoneum	8 (29.6)	13 (54.2)
Bone	2 (7.4)	1 (4.2)
Distant lymph node	8 (29.6)	1 (4.2)
Other	5 (18.5)	3 (12.5)
**Number of metastatic sites,** ***n*** **(%)**		
1	9 (33.3)	8 (33.3)
2	10 (37.0)	6 (25.0)
≥3	8 (29.6)	10 (41.7)
**Sum of diameter (mm), median (range)**	58 (10–188)	58 (17–121)
**Lines of previous systemic treatment,** ***n*** **(%)**		
One	17 (63.0)	11 (45.8)
Two	7 (25.9)	6 (25.0)
Three or more	2 (7.4)	7 (29.2)
Median (range)	1(0*-3)	2(1–4)
**Prior cancer treatment,** ***n*** **(%)**		
Primary tumor resection	18 (66.7)	16 (66.7)
Radiation therapy	4 (14.8)	0
Ablation therapy	13 (48.1)	7 (29.2)
Intra-arterial therapy^#^	12 (44.4)	10 (41.7)
Chemotherapy	26 (96.3)	24 (100.0)

*ECOG* Eastern Cooperative Oncology Group, *PS* performance status

*One patient with BTC had received hepatic arterial infusion of gemcitabine and cisplatin, which is a regional treatment, but was included due to intolerance to chemotherapy. ^#^Intra-arterial therapy includes transarterial infusion chemotherapy and transarterial chemoembolization (TACE)

### Efficacy

In the BTC cohort, 25 patients were evaluable for response, and 7 patients (28.0%; 95% confidence interval [CI]: 12.1-49.4) achieved an objective response, including 2 complete responses (CR) and 5 partial responses (PR). In the PDAC cohort, 20 patients were evaluable for response, and 3 patients (15.0%; 95% CI: 3.2-37.9) achieved an objective response, including 2 CRs and one PR (Table 2). The disease control rate (DCR) was 80.0% (95% CI: 59.3-93.2) and 60.0% (95% CI: 36.1-80.9) for the BTC and PDAC cohorts, respectively. Figure 1c illustrated the best percent of change from baseline in the target lesion size and the best response for evaluable patients in the two cohorts.

**Table 2:** Efficacy outcomes

	BTC cohort (*n* = 27)	PDAC cohort (*n* = 24)
Best overall response^#^, *n* (%)		
CR	2 (8.0)	2 (10.0)
PR	5 (20.0)	1 (5.0)
SD	13 (52.0)	9 (45.0)
PD	5 (20.0)	8 (40.0)
ORR* (CR + PR)		
n (%)	7 (28.0)	3 (15.0)
95% Cl	(12.1–49.4)	(3.2–37.9)
DCR* (CR + PR + SD)		
n (%)	20 (80.0)	12 (60.0)
95% Cl	(59.3–93.2)	(36.1–80.9)
DoR		
Median (95% Cl), months	7.6 (2.2–13.0)	-^†^
PFS		
Median (95% Cl), months	5.1 (2.6–7.6)	2.1 (0.7–3.5)
% at 6 months (95% Cl)	47.9% (31.7–72.6)	20.0% (8.4–47.6)
% at 12 months (95% Cl)	26.2% (13.3–51.6)	13.3% (4.1–43.4)
OS		
Median (95% Cl), months	16.0 (6.1-NE)	5.3 (4.0-6.5)
% at 6 months (95% Cl)	66.1% (50.3–86.9)	44.0% (27.7–69.8)
% at 12 months (95% Cl)	54.4% (38.3–77.3)	39.6% (23.9–65.6)

*PD* progressive disease, *SD* stable disease, *PR* partial response, *CR* complete response, *CI* confidence interval, *ORR* objective response rate, *DCR* disease control rate, *DoR* duration of response, *OS* overall survival, *PFS* progression-free survival, *NE* not evaluable

^#^ Two patients in the BTC cohort and four patients in the PDAC cohort were excluded from the response assessment population due to early discontinuation of treatment before the first scheduled post-baseline imaging assessment. * Responses were assessed in accordance with Response Evaluation Criteria in Solid Tumors version 1.1. ^†^ The median DoR was not reported for the PDAC cohort due to the limited number of responders (*n* = 3)

For the full analysis population, the median follow-up duration was 26.4 months (95% CI 19.6–33.2). Among BTC patients, the median duration of response (mDoR) was 7.6 months (95% CI: 2.2–13.0 months; Table 2 and Supplementary Fig. S1a) and the median PFS (mPFS) was 5.1 months (95% CI: 2.6–7.6 months; Fig. 1d). The median OS (mOS) was 16.0 months (95% CI: 6.1-not estimable [NE]; Fig. 1f), with a 1-year OS rate of 54.4% (95% CI: 38.3%–77.3%; Table 2) and a 2-year OS rate of 48.1% (95% CI: 31.5%–73.5%). Notably, seven patients (26.0%) in the BTC cohort received subsequent systemic treatment post-progression. For the PDAC group, among the three patients who achieved a response, one achieved PR with a DoR of 6.3 months, while the other two achieved CR with DoRs of 9.8 and 14 months, respectively (Supplementary Fig. S1b). However, it should be noted that the patient with a DoR of 14 months died due to an accidental fall from a building while working, unrelated to disease progression. The mPFS was 2.1 months (95% CI: 0.7-3.5 months; Fig. 1e), and the mOS was 5.3 months (95% CI: 4.0-6.5 months; Fig. 1g), with a 1-year OS rate of 39.6% (95% CI: 23.9%-65.6%; Table 2). 4 patients (16.7%) in the PDAC cohort received subsequent systematic treatment post-progression.

**Fig. 2:**
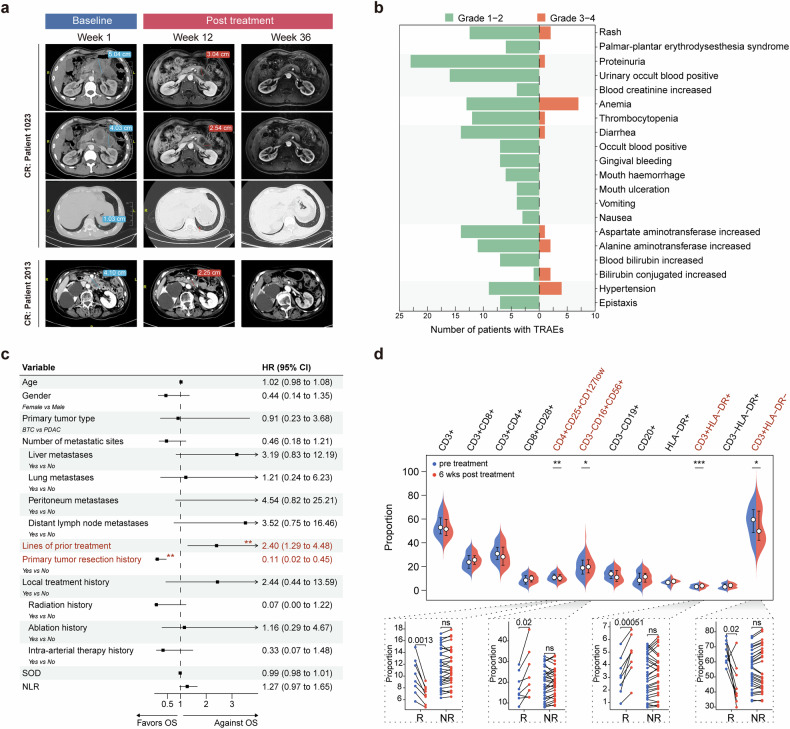
Safety profile and clinicopathologic parameters analysis of the study. **a** Representative magnetic resonance (MR) or computed tomography (CT) images of the complete response (CR) cases in our cohorts. Lesions are marked with horizontal lines indicating their longest target diameters. CT and MR images of CR patient 1023 with PDAC (the top row: metastatic lesion 1 in the peritoneum; the middle row: metastatic lesion 2 in the peritoneum; the bottom row: metastatic lesion in the lung). CT images of CR patient 2013 with BTC (metastatic lesion in the peritoneum). Detailed patient background information can be found in Supplementary Data S1. **b** Overview of treatment-related adverse events (TRAEs) by grade in organ systems with grade 3–4 events. **c** Forest plot of hazard ratios (HR) and 95% confidence intervals (CI) for overall survival (OS) based on various clinicopathological factors in the overall population. Factors favoring OS are shown on the right side of the plot, while factors against OS are shown on the left side. The category of local treatment includes ablation, radiation and intra-arterial therapy. **, *P* < 0.01; SOD, sum of diameter; NLR, neutrophil-to-lymphocyte ratio. **d** Top: comparison of peripheral blood mononuclear cell (PBMC) cluster proportions before treatment (pre-treatment) and 6 weeks post-treatment. *N* = 37 evaluable patients. Statistical significance was determined by Bonferroni’s multiple comparison test. *P < 0.05; **P < 0.01; ***P < 0.001. Bottom: comparison of CD4^+^CD25^+^CD127^low^, CD3^-^CD16^+^CD56^+^, CD3^+^HLA-DR^+^ and CD3^+^HLA-DR^-^ cell cluster proportion pre- and post-treatment split by clinical response. *N* = 8 clinical responders (R), *N* = 29 clinical non-responders (NR). Statistical significance was determined using the Mann-Whitney U test

The CR rates of 8% in BTC and 10% in PDAC observed in our study were notable, considering the rarity of CR cases reported in previous trials of metastatic cancer.^24,25^ A comprehensive characterization of these exceptional responders was summarized in Supplementary Data S1. Representative magnetic resonance or computed tomography images of the CR cases were presented in Fig. 2a and Supplementary Fig. S1c, with the longest DoR exceeding 3 years (Supplementary Data S1).

### Safety

At the time of data cutoff, the median duration of exposure was 2.4 months (range, 0.1–41.0 months) for the study treatment (SHR-1701: median, 1.5 months; range, 0.03–26.4 months; famitinib: median, 2.1 months; range, 0.1–41.0 months). The majority of patients (88.2%) experienced at least one treatment-related adverse event (TRAE). The most common any-grade TRAEs were proteinuria (47.1%), anemia (39.2%), and positive urinary occult blood (31.4%; Supplementary Table S1). Grade 3 or 4 TRAEs occurred in 15 patients (29.4%; Fig. 2b), with the most common being anemia (13.7%) and hypertension (7.8%). In addition, 16 cases (31.4%) experienced potential immune-related adverse events, with the most common being rash (23.5%) and hypothyroidism (13.7%; Supplementary Table S2). Among them, 2 cases (3.9%) were grade 3-4. A total of 8 subjects received immunosuppressive therapy, with 4 cases of topical corticosteroids for skin toxicity. No grade 5 TRAEs were reported.

TRAEs led to treatment interruption and treatment discontinuation of SHR-1701 in 7 (13.7%) and 8 (15.7%) patients, respectively. Regarding famitinib, TRAEs resulted in 17 (33.3%) treatment interruption, 11 (21.6%) dose reduction, and 5 (9.8%) treatment discontinuation cases. Overall, 4 patients (7.8%) discontinued all treatment due to TRAEs.

### Comprehensive clinicopathologic features associated with treatment benefit

Multivariate analysis demonstrated that in the overall population, patients who had previously undergone primary tumor resection exhibited longer OS (HR = 0.11, 95% CI = 0.02–0.45; Fig. 2c and Supplementary Fig. S1d), while a higher number of lines of previous systemic treatment was identified as a risk factor for OS (HR = 2.4, 95% CI = 1.29–4.48; Fig. 2c). Additionally, distant lymph node metastases and neutrophil-to-lymphocyte ratio (NLR) were identified as risk factors for PFS (Supplementary Fig. S1e and S1f).

The presence or absence of common metastases, including liver, lung, peritoneum, and distant lymph nodes, did not show a significant correlation with treatment response (Supplementary Fig. S1g). Similarly, PD-L1 expression (Supplementary Fig. S1h) and microsatellite stability (MSS) status (Supplementary Fig. S1i) did not correlate with treatment response in our cohort. However, baseline carcinoembryonic antigen (CEA) levels were significantly higher in BTC non-responders (Supplementary Fig. S1j), and baseline carbohydrate antigen 19-9 (CA 19-9) levels were higher in PDAC non-responders (Supplementary Fig. S1k).

In the post hoc analysis, we investigated the association between peripheral blood immunophenotype and clinical outcome by collecting peripheral blood mononuclear cells (PBMCs) from participants before treatment and 6 weeks post-treatment. Our analysis identified revealed notable changes in several cell subtypes, implying that the combination of SHR-1701 and famitinib treatment markedly improved the tumor microenvironment (Fig. 2d). The decrease in CD4^+^CD25^+^CD127^low^ regulatory T cells (Tregs) signified a decrease in immunosuppressive function,^26^ while the increase in CD3^-^CD16^+^CD56^+^ NK cells suggested an increase in cytotoxic activity against tumors.^27^ Additionally, the increase in CD3^+^HLA^-^DR^+^ activated T cells and the decrease in CD3^+^HLA-DR^-^ cells (Fig. 2d) suggested a shift towards a more activated T cell subset.^28^ Interestingly, these consistent alterations were predominantly observed in responders, implying a more favorable immune microenvironment for anti-tumor responses, whereas non-responders exhibited varied changes. These findings suggested that changes in peripheral blood immunology within 6 weeks after treatment may be indicative of treatment efficacy and could potentially serve as a predictive biomarker in the context of SHR-1701 plus famitinib therapy, warranting further validation in larger clinical trials.

### Distinct immune and metabolic profiles associated with treatment response

**Fig. 3:**
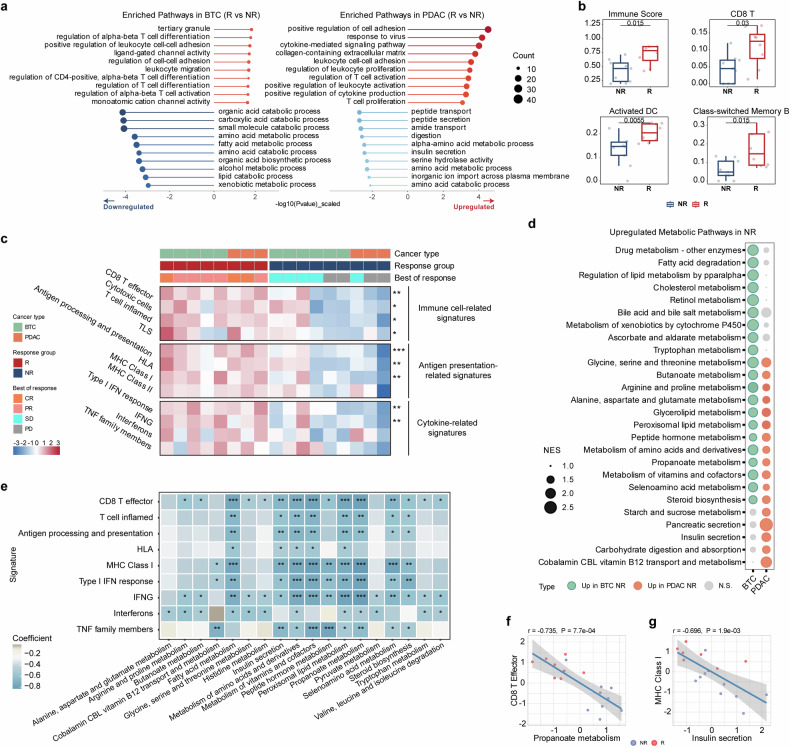
Immune and metabolic profiles associated with treatment response. **a** Pathway enrichment analysis of differentially expressed genes between responders and non-responders in BTC (left) and PDAC (right) cohorts. Red for upregulated pathways in responders and blue for downregulated ones. **b** Comparison of the estimated abundance of immune cell types in the tumor microenvironment (TME) between responders (R) and non-responders (NR) using xCell deconvolution analysis. Statistical significance was determined using the Mann-Whitney U test. **c** Heatmap displaying the enrichment scores of 12 gene set signatures in responders (R) and nonresponders (NR) across BTC and PDAC cohorts. The signatures were categorized into three main groups: immune cell-related signatures, antigen presentation-related signatures, and cytokine-related signatures. Statistical significance was determined using the Mann-Whitney U test. *, *P* < 0.05; **, *P* < 0.01; ***, *P* < 0.001; CR, complete response; PR, partial response; SD, stable disease; PD, progressive disease. **d** Bubble plot showing upregulated metabolic pathways in non-responders (NR) compared to responders (R) in BTC (red) and PDAC (blue) cohorts. Bubble size represented the normalized enrichment score (NES). N.S. (gray) denoted non-significant (*P* value ≥ 0.05) pathways. **e** Correlation matrix showing the interplay between NR-high metabolic pathways and immune signatures. Color scale represented Spearman correlation coefficient. Statistical significance was determined using the Mann-Whitney U test. *, *P* < 0.05, **, *P* < 0.01, ***, *P* < 0.001. **f** Scatter plot illustrating the negative correlation between the peroxisomal lipid metabolism pathway score and the CD8^+^ T cell effector signature score in non-responders (NR, blue) and responders (R, red). Spearman correlation coefficient (r) = -0.735, *P* = 7.7e-04. **g** Scatter plot demonstrating the negative correlation between the insulin secretion pathway score and the MHC class I signature score in non-responders (NR, blue) and responders (R, red). Spearman correlation coefficient (r) = -0.696, *P* = 1.9e-03

RNA-seq data of baseline primary tumor samples revealed distinct gene expression patterns between BTC and PDAC patients (Supplementary Fig. S2a). Despite cancer type-specific mechanisms, differentially expressed genes (Supplementary Fig. S2b) and pathway enrichment analysis identified common features related to treatment efficacy in both responders and non-responders (Fig. 3a; Supplementary Data S2). Responders exhibited upregulation of immune-related pathways, including leukocyte adhesion, migration, and activation, as well as T cell differentiation and activation. In contrast, non-responders showed enrichment of metabolic pathways, such as amino acid metabolism and catabolism, implying the potential role of heightened metabolic activity in treatment resistance.

To elucidate differences in immune cell recruitment and activation, we estimated the abundance of diverse immune cell types in the TME using deconvolution analysis and scored 12 gene set signatures previously reported to be associated with immunotherapy efficacy for each patient (Supplementary Data S3). Our analysis revealed a higher level of overall immune cell infiltration in the responder group (*P* = 0.015), particularly in the proportions of CD8^+^ T cells (*P* = 0.03), activated dendritic cells (*P* = 0.006), and class-switched memory B cells (*P* = 0.015; Fig. 3b and Supplementary Fig. S2c). Moreover, responders harbored significantly elevated scores in most signatures related to immune cell activation, antigen presentation, and cytokine activity (Fig. 3c).

Counterintuitively, we observed elevated levels of TGFB1 expression and TGF-β signaling in responders of our cohort (Supplementary Fig. S2d), despite their usual association with poor responses as reported. Moreover, genes and pathways related to angiogenesis and hypoxia were also upregulated in responders (Supplementary Fig. S2e-f), further supporting the notion that SHR-1701 plus famitinib might help overcome these potentially immunosuppressive factors, thereby enhancing immunotherapy efficacy.^29,30^

### Metabolic reprogramming in treatment resistance and its interplay with the immune landscape

Considering the complexity of tumor metabolic networks, we employed gene set enrichment analysis (GSEA) to comprehensively profile the upregulated metabolic pathways in patients with poor response (Supplementary Data S4). Certain pathways consistently elevated in non-responders across both cancer types were involved in the metabolism of amino acids (glycine and arginine, etc.), lipids (such as butanoate, propanoate, and steroid biosynthesis), and vitamins, reflecting a broad enhancement of metabolic processes in treatment-resistant patients (Fig. 3d and Supplementary Fig. S2g-h). Additionally, we also observed cancer type-specific preferences in upregulated metabolic modules. In BTC non-responders, the predominantly upregulated pathways were related to drug metabolism, fatty acid degradation, and bile acid metabolism, suggesting a focus on detoxification and lipid handling; in contrast, PDAC-specific active processes were associated with carbohydrate metabolism and pancreatic functions, underlining the unique metabolic demands of pancreatic tumors.

To uncover the interplay between metabolic reprogramming and the immune landscape, we calculated scores for the top non-responder-enriched (NR-high) metabolic pathways and analyzed their correlation with immune signatures. Remarkably, several commonly upregulated metabolic pathways, such as peroxisomal lipid metabolism, propanoate metabolism, and the metabolism of vitamins and cofactors, exhibited strong negative correlations with CD8^+^ T cell effector and MHC class I signatures, suggesting that their upregulation might shape an immunosuppressive microenvironment, thereby hindering patients’ responses to immune-based therapy like SHR-1701 (Fig. 3e, f). Interestingly, as an integral part related to cellular metabolism, insulin secretion pathways also displayed negative correlations with immune signatures (Fig. 3g).

### Immune/metabolism score as a potential predictive biomarker for immunotherapy response

**Fig. 4:**
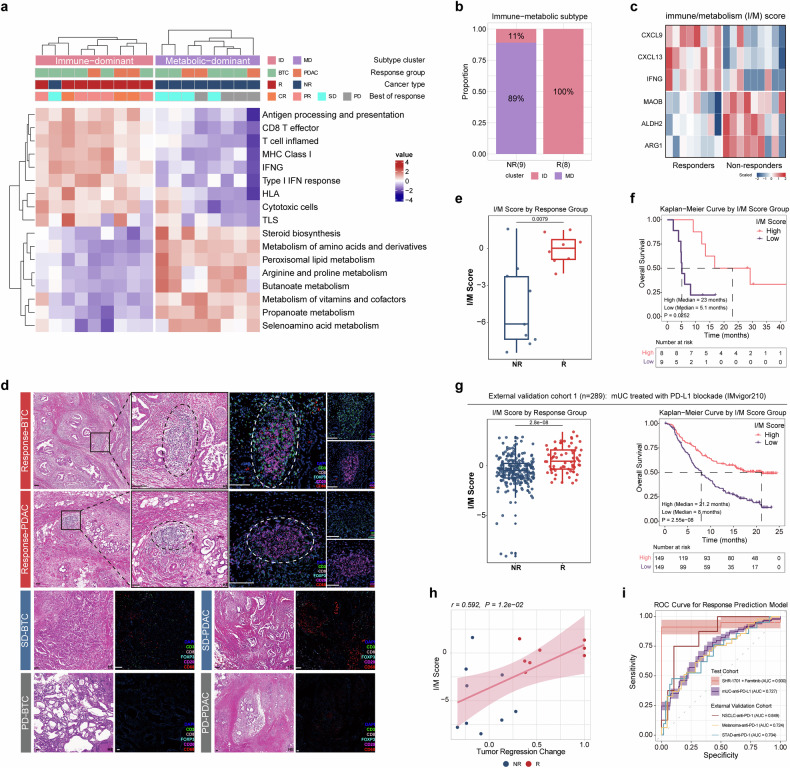
Immune/metabolism score as a potential predictive biomarker for immunotherapy response. **a** Heatmap showing the ssGSEA scores of immune signatures and metabolic pathways in patients stratified into ‘immune-dominant’ (left, pink) and ‘metabolic-dominant’ (right, purple) subgroups. The color scale represents the normalized expression values. **b** Proportion of patients in the immune-dominant (ID) and metabolic-dominant (MD) subgroups within the non-responder (NR) and responder (R) groups. **c** Heatmap displaying the expression levels of the six genes used to calculate the immune/metabolism (I/M) score in responders and non-responders. **d** Representative mIHC images of tumor samples from patients with different BOR in BTC and PDAC patients. Color-coded for immune cell populations was as illustrated, with the TLS region circled by a dashed line. The scale bar at the bottom left corner of the mIHC images represents 0.1 mm. TLS, tertiary lymphoid structure; SD, stable disease; PD, progressive disease. **e** Comparison of I/M scores between non-responders (NR) and responders (R; *P* = 0.0079, Mann-Whitney U test). **f** Kaplan-Meier curves for overall survival (OS) stratified by I/M score group (high vs. low), with median OS and *P* value (0.0252, log-rank test). The number at risk table was included. **g** External validation cohort 1 (*n* = 289, IMvigor210): I/M scores in R vs. NR (left; *P* = 2.8e-08, Mann-Whitney U test), and OS stratified by I/M score group (high vs. low) with median OS, *P* value (2.55e-08, log-rank test), and number at risk table (right). mUC, metastatic urothelial carcinoma. **h** Scatter plot showing the correlation between I/M score and tumor regression change in non-responders (NR, blue) and responders (R, red). Spearman correlation coefficient (r) = 0.592, with a *P* value of 1.2e-02. **i** Receiver operating characteristic (ROC) curve for the response prediction model using the I/M score. The plot showed the performance of the model in the test cohorts (SHR-1701 plus famitinib, AUC = 0.930; mUC-anti-PD-L1, AUC = 0.727) and three external validation cohorts. AUC area under the curve, mUC metastatic urothelial carcinoma, NSCLC non-small cell lung cancer, STAD stomach adenocarcinoma

Next, based on selected immune-metabolic features most strongly associated with treatment response, we stratified patients into two subgroups via clustering algorithms: ‘immune-dominant’ (ID) and ‘metabolic-dominant’ (MD). The ID subgroup showed higher levels of immune cell infiltration, activated immune-related phenotypes, and lower metabolic activity (Fig. 4a), whereas the MD subgroup displayed an enriched metabolic pattern with immune dysfunction. Notably, patients of the ID subtype demonstrated better clinical response rates (Fig. 4b) in our cohort. Applying the same strategy to independent, published immunotherapy cohorts resulted in a similar stratification pattern, with ID subtype patients experiencing greater clinical benefits than MD subtype patients (Supplementary Fig. S3a-c).

To enhance clinical practicality, we developed an immune/metabolism (I/M) score to predict immunotherapy efficacy, relying on the pretreatment expression of only six genes (Fig. 4c; Supplementary Fig. S4a). Of particular interest, CXCL13, CXCL9, and IFNG were key genes involved in the tertiary lymphoid structures (TLS) formation, consistent with the increased proportions of dendritic cells and B cells in responders as previously mentioned (Fig. 3b). Multiplex immunofluorescence (mIHC) further confirmed the presence of TLS in responders, while patients with stable diseases (SD) had mainly myeloid cell infiltration, and progression disease (PD) patients displayed an immunologically “cold” tumor state (Fig. 4d and Supplementary Fig. S4b). These findings revealed a contribution of TLS to BTC/PDAC immunotherapy and indicated the biological TME basis of the I/M score.

A markedly higher level of I/M score was observed in responders versus nonresponders (Fig. 4e). Moreover, patients with higher scores had remarkably longer OS (Fig. 4f) and PFS (Supplementary Fig. S4c), which positively correlated with pathological tumor regression change as well (Fig. 4h). Validation of the I/M score in various advanced cancer types treated with PD-(L)1 inhibitors confirmed that higher scores were consistently associated with better treatment response and prolonged OS (Fig. 4g and Supplementary Fig. S4d-f).

Leveraging machine learning (Supplementary Fig. S4g), I/M scores showed promising predictive power in both our cohort (area under the receiver operating characteristic curve [AUC] of 0.930) and multiple external cohorts of pan-cancer (Fig. 4i), supporting its utility as a potential effective immunotherapy biomarker.

## Discussion

For patients with refractory BTC and PDAC, the shortage of effective antitumor regimens has long been a conundrum for oncologists.^31^ To our knowledge, this is the first reported study to investigate the combination of a bispecific inhibitor targeting TGF-β/PD-L1 and a TKI in solid tumors, representing a novel approach to address this unmet need. Our findings suggest that SHR-1701 plus famitinib achieves promising efficacy in patients with advanced BTC or PDAC who failed previous treatment, with an ORR of 28% in the BTC cohort and 15% in the PDAC cohort, including confirmed CRs in 8% and 10% of patients, respectively. The median OS for BTC patients was 16.0 months, comparable to first-line treatment. For PDAC patients, the mOS was 5.3 months, which is notable for a chemotherapy-free regimen despite most patients in this cohort having been heavily treated with ≥2 lines of therapy.

In the ongoing exploration of novel immunotherapies, several studies have shown potential benefits.^32,33^ The LEAP-005 Phase II trial reported an ORR of 10% and a mOS of 8.6 months with lenvatinib and pembrolizumab in advanced BTC patients.^34^ Likewise, sintilimab and anlotinib in second-line treatment achieved a mOS of 12.3 months.^35^ In contrast, the immunotherapy landscape for PDAC is less optimistic, with more trials switching to evaluating the chemotherapy-dominated immunotherapy combinations (NCT04803851, NCT05493995), yet the outcomes remain challenging.^36^

The renaissance in antibody-based therapies has focused on developing bispecific and even tri-specific antibodies, aiming to offer a more accessible and effective approach.^37,38^ Preclinical data for SHR-1701 demonstrated its bifunctional design advantage, delivering TGF-β trap specifically to the tumor site via PD-L1 binding, reducing systemic adverse effects and boosting TGF-β neutralization in TME.^39–43^ Despite the appealing concept, the clinical performance of bispecific antibody monotherapies has fallen short of expectations, as seen in phase III trials of bintrafusp alfa (with an ORR of 10% in BTC),^40,44^ highlighting the need for optimization of drug design and exploration of rational combination strategies. Our study exemplified that pairing bispecific antibodies with anti-angiogenic agents, when the safety profile allows, may maximize therapeutic efficacy by creating a more favorable microenvironment for immune-mediated tumor destruction.^45,46^

Building upon the encouraging clinical benefits, the safety profile of SHR-1701 plus famitinib was manageable. The most common TRAEs included proteinuria, anemia, and positive urinary occult blood, consistent with previous ICI and anti-angiogenic therapy studies.^34^ Hematologic toxicities were the predominant grade 3 or higher AEs, aligning with the known tolerability of SHR-1701.^17^ Despite four patients discontinuing treatment due to TRAEs, no grade 5 TRAEs or treatment-related deaths were reported.

Several baseline characteristics were associated with prognosis, with prior primary tumor resection being a key factor of improved OS, which underscores the impact of reducing the overall tumor burden as early as possible. In line with previous reports, lower baseline levels of CEA or CA 19-9, as well as shifts in peripheral blood immunophenotype such as decreased Tregs and increased NK cells and activated T cell subsets,^47^ were correlated with improved therapeutic outcomes. Given that most of these analyses were not prespecified, interpretation of the results should be approached with caution.

Previous experimental studies have fostered optimism, suggesting that blocking TGF-β may even render immune-excluded tumors responsive to ICIs.^48^ However, our RNA-seq data reveal that the preexisting immune infiltration remains a major determinant of the anti-PD-L1/TGF-β response.^49–51^ Baseline primary tumor samples of responders showed enrichment of immune-related signatures, and increased densities of TLS. Notably, CR patients in our cohort exhibited strong baseline immune-related signatures and relatively low NLR (0.83-2), despite their heterogeneous primary tumor histology and tumor burden (Supplementary Data S1), suggesting that a favorable immune microenvironment may be crucial for achieving complete responses in our clinal setting. Nevertheless, the limited number of CR cases precludes definitive conclusions about the factors driving the transition from PR to CR.

Intriguingly, responders in our cohort did exhibit higher levels of TGF-β and angiogenesis pathways, despite their usual association with poor outcomes as reported. These findings further support the rationale for combining SHR-1701 and famitinib, indicating that this combo can synergize to overcome some resistance mechanisms, which might explain the higher CR rates and improved survival observed in our trial. However, while effective against TGF-β and VEGF signaling, this combination may not fully counteract the immunosuppressive effects driven by elevated metabolic activity, as indicated by the enrichment of certain metabolic pathways in non-responders.^52,53^ Metabolic reprogramming in tumors not only fuels rapid progression but also competes with immune cells for nutrients, hindering the effectiveness of immunotherapies. Ongoing trials are exploring amino acid-restricted diets, metformin targeting insulin secretion, or statins modulating lipid metabolism (NCT03889795, NCT03889795) as adjunct therapies for cancer patients.^52,54–56^

Given the complexity of TME and the need for predictive biomarkers, we developed the I/M score to identify patients most likely to respond to the combination of SHR-1701 and famitinib. This score stands out from other transcriptomic signatures by pioneering the integration of both immune and metabolic features, capturing a panel of key genes related to immune activation (CXCL13, CXCL9, IFNG) and metabolic pathways (MAOB, ALDH2, ARG1) into a single metric.^50^ Of note, inhibitors targeting these metabolic genes are under investigation in clinical trials, and combining metabolic therapies with SHR-1701 and famitinib might offer a chance to rescue the non-responsive patient subsets.^54^ This model demonstrates predictive performance across various cancer types, as evidenced by the association of higher I/M scores with improved responses and survival in our cohort and external pan-cancer cohorts. Yet, to confirm the I/M score’s superior predictive value and practical utility in guiding immunotherapy treatment decisions, head-to-head comparisons with established signatures are essential.

The study has several limitations that should be acknowledged. The single-center design may introduce selection bias and limit generalizability, while the lack of a control group in this phase II trial might weaken the reliability of evidence. The calculation of ORR, PFS, and OS could also be impacted by selection bias because of the relatively small sample size. Although our data provide preliminary evidence supporting the safety of SHR-1701 combined with famitinib, further studies with longer treatment duration and larger patient cohorts are needed to fully establish the long-term safety profile of this combination therapy. Moreover, the limitations of detection timepoints and methods should be considered, as only baseline RNA samples may not fully reflect dynamic changes during treatment. The unavailability of public immunotherapy datasets for BTC and PDAC precludes external validation in the same cancer types, and the limited number of retrospectively collected specimens results in insufficient biomarker analysis. These factors may have influenced the ability to detect significant associations between biomarkers and treatment response, limiting the generalizability of our findings. Lastly, given the lack of comparable clinical trials investigating PD-L1/TGF-β inhibition with TKI therapy, future research is warranted to validate these findings in larger, prospective clinical trials. With the rapid advancements in immuno-oncology and the increasing availability of high-throughput technologies, we expect to see a more granular understanding of tumor-immune interactions and the identification of novel therapeutic targets.

In summary, our study uncovers the potential of SHR-1701 plus famitinib as an effective and safe subsequent-line therapy for refractory BTC and PDAC, providing proof-of-concept data supporting triple blockade of PD-L1, TGF-β and angiogenesis pathways in managing these challenging malignancies. This combination strategy may serve as a promising alternative for patients who have not responded to or are not suitable for standard therapy. Furthermore, the identification of predictive biomarkers, including the I/M score, may guide patient selection and contribute to personalized cancer care.

## Materials and methods

### Study design and patients

This open-label, single-arm and two-stage Simon-designed trial was conducted in Shanghai, China. The trial aimed to evaluate the efficacy and safety of SHR-1701 in combination with famitinib in treating advanced BTC and PDAC.

Eligibility criteria included an age range of 18–75 years, a histologically or cytologically confirmed diagnosis of advanced pancreatic or biliary tract cancer, failure of at least one line of prior therapy or intolerance to chemotherapy, an expected survival of ≥3 months, and adequate organ and bone marrow function. Key exclusion criteria included previous treatment with ICIs such as PD-1, PD-L1, CTLA4, and TGF-β inhibitors, known allergies to study drugs, and serious cardiac arrhythmia, diabetes, or serious active infection or other illness that would preclude study participation. Complete details on inclusion and exclusion criteria can be found in the study protocol.

The study protocol and all amendments were approved by the Institutional Review Board (Approval ID: 2006219-18-2011 A). All participants provided written informed consent for study participation and specimen collection. The study was done following Good Clinical Practice guidelines and the Declaration of Helsinki.

### Ethics approval and consent to participate

The study was registered in the Chinese Clinical Trials Registry Platform (ChiCTR2000037927) and approved by the Ethics Committee of Fudan University Shanghai Cancer Center (Approval ID: 2006219-18-2011 A). Written informed consent was obtained from each patient.

### Procedures

Participants received SHR-1701 at a dose of 30 mg/kg intravenously every 3 weeks in combination with oral famitinib (Fig. 1a), which was initiated at 20 mg daily and could be adjusted to 15 mg in the event of toxicity. Treatment was administered in 21-day cycles and continued until disease progression, intolerable toxicity, voluntary withdrawal of consent, or any other condition necessitating discontinuation as judged by the investigator. If disease progression per the Response Evaluation Criteria in Solid Tumors (RECIST) version 1.1 occurs, subjects may continue study treatment at the investigator’s discretion if there is potential clinical benefit, acceptable safety, and patient consent.

In this study, tumor response assessment was performed every 6 weeks using radiographic imaging and evaluated per RECIST 1.1. Safety profiles, including the incidence, severity, and causality of AEs, were monitored and AEs were graded according to the Common Terminology Criteria for Adverse Events (CTCAE) version 5.0. Physical examinations and routine laboratory tests were performed regularly at the end of each cycle to evaluate treatment response and patient safety.

### Endpoints

In this phase II study, the primary endpoint was objective ORR, defined as the proportion of patients achieving confirmed complete or PR per RECIST v1.1, among those who received at least one cycle of the study treatment. Secondary endpoints included DCR, PFS, OS, and safety evaluation through AE monitoring. Additionally, the study aimed to explore the correlation between potential biomarkers in tumor tissue or peripheral blood and treatment efficacy to identify predictive markers for response.

### Statistical analysis

Simon’s 2-stage design was used to assess ORR based on radiologic assessment, with the null hypothesis being an ORR of 10%, and the alternative hypothesis was an ORR of 30%, with a one-sided alpha of 5% and 80% power. In stage 1, 15 patients were enrolled, and if ≥2 responses were observed, an additional 10 patients would be accrued in stage 2, requiring ≥5 responses among 25 patients to consider the treatment promising.

The evaluable population for response assessment comprised patients who received at least one dose of the study treatment and had at least one post-treatment tumor assessment. Safety analyses included all patients who received at least one dose of the study treatment. ORR and DCR were calculated with 95% Clopper-Pearson CIs. Survival analysis was performed using the Kaplan-Meier method, with medians and two-sided 95% CIs provided when applicable. AEs were summarized using standard frequency tables (number and percentages of patients with events). The hazard ratio for survival was estimated from Cox analysis models. Statistical significance was determined by performing a Mann-Whitney U test to compare the two groups. The correlation analysis was conducted using the Spearman correlation method. A P-value less than 0.05 was considered statistically significant.

### Sample collection and RNA-seq data processing

Formalin-fixed, paraffin-embedded (FFPE) tissue samples were collected at baseline from 17 primary tumors: 11 BTC (5 responses [R], 6 non-responses [NR]) and 6 PDAC (3 R, 3 NR). Total RNA was extracted from the FFPE samples using the RNeasy FFPE Kit (Qiagen), following the manufacturer’s protocol. The quality and quantity of the extracted RNA were assessed using a NanoDrop spectrophotometer and an Agilent 2100 Bioanalyzer. Samples with an RNA integrity number (RIN) ≥ 7 were considered suitable for RNA sequencing.

RNA sequencing libraries were prepared using TruSeq RNA Library Prep Kit (Illumina), according to the manufacturer’s instructions. The libraries were sequenced on Illumina HiSeq 2500 with 100 bp paired-end reads at a depth of 50 million reads per sample. Trimmomatic was used to treat the raw sequencing data in order to eliminate adapters and poor-quality reads. The STAR aligner was then used to match the cleaned reads to the human reference genome (hg38). The aligned reads were quantified at the gene level using HTSeq-count. The raw count data were then normalized and log-transformed using the trimmed mean of M-values (TMM) normalization.

### Differential expression gene and pathway enrichment analysis

Analysis of differential expression genes (DEGs) was carried out in R using the DESeq2 package (v1.42.0). Up-regulated DEGs (R vs NR) were defined as genes with adjusted P-value (padj) < 0.05 and log2FC > log2(1), while down-regulated DEGs had padj < 0.05 and log2FC < log2(1). Pathway enrichment analysis was performed separately for the up-regulated and down-regulated DEGs using the enrichGO function from the clusterProfiler package (v4.10.0). The significance cutoff for the enrichment analysis was set to a padj and P-value of 0.05.

### Immune deconvolution analysis

We performed deconvolution analysis using the xCell package (v1.1.0) to estimate the immune cell composition of our samples. xCell, a widely used computational method, was chosen for its comprehensive coverage of cell subtypes and extensive validation in the field. The analysis was applied to the gene expression matrix, yielding the estimated fractions of various immune cell types for each sample.

### Signature score calculation

The gsva function from the GSVA package (v1.50.0) was applied to the expression matrix and the signature list to calculate ssGSEA enrichment scores for each gene set in each sample, allowing for the assessment of gene set activity at the individual sample level. The 12 gene set signatures previously reported to be associated with immunotherapy efficacy were obtained from the IOBR package (v0.99.9) and published literature, with detailed information summarized in Supplementary Data S3, illustrating their widespread application to evaluate immunotherapy response and prognosis. The metabolism genesets enriched in non-responders and other geneset signatures used in the results were summarized in Supplementary Data S3 as well.

GSEA

GSEA was performed using the clusterProfiler package (v4.10.0), which leverages the fgsea algorithm for fast GSEA implementation. The analysis was comprehensively conducted using metabolic pathways of the Kyoto Encyclopedia of Genes and Genomes (KEGG) and REACTOME with 1000 permutations (Supplementary Data S4). Pathways with a normalized enrichment score (NES) > 1 and a *P* value < 0.05 were considered significantly enriched.

### Semi-supervised stratification

The stratification was performed using the tme_cluster function from the IOBR package (v0.99.9), which applies clustering algorithms to group patients based on selected immune-metabolic features. The following were the selection criteria used for these features: Immune characteristics that demonstrated significant variations (*P* < 0.05) among the responder and non-responder populations were taken into consideration. We chose the metabolic features that showed the strongest negative relationships (*P* < 0.05) with most of the immunological markers. The optimal number of clusters was determined using the NbClust package (v3.0.1), and the patients were partitioned into the determined number of clusters using k-means clustering method.

### I/M score

Univariate logistic regression analysis was performed using the glmnet package (v4.1.4) to explore the predictive value of candidate genes. Genes that showed no less than a onefold difference in expression across responders and non-responders and that fit into the immune-metabolic parameters utilized for patient classification were chosen as candidates. Finally, an immune-metabolic (I/M) score was calculated for each sample using the regression coefficients of the top 6 genes with the most significant P-values. The samples were then grouped into high and low I/M score groups based on the median score for further analysis. Details on the independent pan-cancer immunotherapy cohorts used for validation were provided (Fig. 4g and Supplementary Fig. S4d-f).

### mIHC

We used antibodies targeting CD3 (Abcam; catalog no. ab135372), CD8 (CST; catalog no. #98941), CD20 (Abcam; catalog no. ab64088), FOXP3 (Servicebio; catalog no. GB112325), and CD68 (Servicebio; catalog no. GB113109) to perform immunostaining of TLS. The slides were scanned using the PerkinElmer Vectra3 platform.

### Prediction model development

Machine learning algorithms were employed to assess the predictive power of the I/M score using mlr3verse package (v0.2.8). We evaluated multiple machine learning algorithms, including logistic regression, linear discriminant analysis (LDA), support vector machine (SVM), naive Bayes, k-nearest neighbors (KNN), decision tree, and random forest, using a repeated cross-validation resampling strategy. The repeated cross-validation resampling strategy ensured robust model validation, which involved dividing the data into multiple folds and repeating the process to obtain reliable performance estimates. The performance of each algorithm was assessed using a range of metrics, including area under the receiver operating characteristic curve (AUC), accuracy, sensitivity, specificity, false negative rate (FNR), and false positive rate (FPR). Based on the AUC score, naive Bayes was selected as the final model (Supplementary Fig. S4g), trained on the entire training set, and subsequently tested on multiple external independent datasets to assess its generalizability.

### Data visualization

The following R packages were used for visualization: ggplot2 (v3.4.2), ggrepel (v0.9.3), ggpubr (v0.6.0), ggsignif (v0.6.4), ComplexHeatmap (v2.18.0), and IOBR (v0.99.9).

## Supplementary information

Study Protocol

Supplementary Materials

Supplementary Data S1

Supplementary Data S2

Supplementary Data S3

Supplementary Data S4

## Data Availability

The RNA-seq data generated in this study have been deposited in the NCBI Sequence Read Archive under BioProject accession number PRJNA1173873. All other data supporting our findings of this work are available from the corresponding authors upon reasonable request.
