# Supplementary material for: Clinical and biomarker analyses of SHR-1701 combined with famitinib in patients with previously treated advanced biliary tract cancer or pancreatic ductal adenocarcinoma: a phase II trial
Source: Signal Transduct Target Ther. 2024 Dec 13;9:347. doi: 10.1038/s41392-024-02052-3 (PMC11638339; doi:10.1038/s41392-024-02052-3)
Supplement: Supplementary file 1 — Study Protocol [file 41392_2024_2052_MOESM1_ESM.pdf]

Exploratory Study of SHR-1701 in Combination with Famitinib for the Treatment of Advanced Pancreatic Cancer and Biliary Tract Tumors

Protocol Number:	PANC-BTC-IIS-SHR1701-FMTN
Protocol Version:	V 2.1
Protocol Version Date:	September 15, 2021
Principal Investigators:	Professor Zhiqiang Meng / Jing Xie
Clinical Research Institution:	Fudan University Shanghai Cancer Center

Protocol Abstract

**Title**	Exploratory Study of SHR-1701 in Combination with Famitinib for the Treatment of Advanced Pancreatic Cancer and Biliary Tract Tumors
**Identifier**	PANC-BTC-IIS-SHR1701-FMTN
**Version/Date**	V2.1 / September 15, 2021
**Researchers**	Fudan University Shanghai Cancer Center Zhiqiang Meng / Jing Xie
**Study population**	Advanced pancreatic cancer or biliary tract tumors that have failed ≥1 lines of treatment or are intolerant to systemic therapy toxicity
**Study Design**	Single-arm, single-center, Simon's two-stage exploratory study
**Number of Subjects**	60
**Objective**	Evaluation of the efficacy and safety of SHR-1701 in combination with famitinib for the treatment of advanced pancreatic cancer and biliary tract tumors
**Endpoint**	**Primary Endpoint:**<br>Objective Response Rate (ORR) based on RECIST 1.1 criteria.<br>**Secondary Endpoints:**<br>(1) Efficacy Measures:<br>1. Disease Control Rate (DCR) based on RECIST 1.1 criteria.<br>2. Progression-Free Survival (PFS) based on RECIST 1.1 criteria.<br>3. Overall Survival (OS).<br>4. Quality of Life (QoL) assessed using the EORTC QLQ-C30 questionnaire.<br>(2) Safety Measures:<br>Including vital signs, laboratory parameters, Adverse Events (AE), Serious Adverse Events (SAE), drug-related AE and SAE.<br>(3) Biomarker Exploration.
**Procedures**	This study adopts a Simon's two-stage design. Subjects are administered SHR-1701 (30mg/kg, intravenous, once every 3 weeks) in combination with famitinib (20mg, orally, once daily). The initial famitinib dose may be adjusted to 15mg based on the subject's previous treatment tolerance and

current condition. Treatment is administered in cycles of three weeks each, continuously until a treatment termination criterion is met or the subject withdraws from the study. If disease progression per RECIST 1.1 occurs, subjects may continue study treatment at the investigator's discretion if there is potential clinical benefit, acceptable safety, and patient consent.

In Simon Stage I, 15 subjects with pancreatic cancer and 15 subjects with biliary tract tumors are enrolled. If Stage I shows more than 1 case of complete response (CR) or partial response (PR), the study proceeds to Stage II; otherwise, the study is terminated. In Stage II, enrollment continues until reaching approximately 30 subjects, to ensure at least 25 evaluable subjects after accounting for potential drop-out. If there are more than 5 cases of PR or CR in Stage II, the treatment is deemed effective; otherwise, it is considered ineffective.

Inclusion and exclusion criteria

Inclusion criteria:

1) Voluntarily participate and sign the informed consent form;
2) Age between 18 and 75 years, any gender;
3) Histologically or cytologically confirmed pancreatic cancer or biliary tract tumor (TNM stage: stage IIIb/IV); failure of ≥1 line of treatment or intolerance to systemic therapy toxicity; presence of at least one measurable lesion based on Response Evaluation Criteria in Solid Tumors (RECIST) version 1.1;
4) Expected survival period of ≥3 months;
5) Eastern Cooperative Oncology Group (ECOG) performance status of 0-1;
6) Sufficient organ and bone marrow function, defined as follows:
    a) Absolute neutrophil count (ANC) ≥ 1,500/mm^3 (1.5 × 10^9/L);
    b) Platelet count (PLT) ≥ 100,000/mm^3 (100 × 10^9/L);
    c) Hemoglobin (Hb) ≥ 9 g/dL (90 g/L);
    d) Serum albumin ≥ 2.8 g/dL;
    e) Serum creatinine ≤ 1.5 times the upper limit of normal (ULN) or creatinine clearance ≥ 50 ml/min;
    f) Total bilirubin (TB) ≤ 1.5 × ULN, or TB > 1.5 × ULN with direct bilirubin (DBil) ≤ 1 × ULN for liver metastasis patients or liver cancer patients, which should be ≤ 2 × ULN;

g) Aspartate aminotransferase (AST/SGOT) or alanine aminotransferase (ALT/SGPT) levels ≤ 2.5 × ULN, or ≤ 5 × ULN for liver metastasis patients or liver cancer patients;

h) Left ventricular ejection fraction (LVEF) ≥ 50%; QTc < 450 ms for males, < 470 ms for females;

7) Patients not receiving anticoagulant therapy should have international normalized ratio (INR) ≤ 1.5 and activated partial thromboplastin time (APTT) ≤ 1.5 times the upper limit of normal. Patients receiving full-dose or extra-gastrointestinal anticoagulant therapy can enter the clinical trial if the anticoagulant dose has been stable for at least 2 weeks before entering the study and the coagulation test results are within the range allowed by local treatment restrictions;

8) Women of childbearing potential must undergo a pregnancy test within 2 weeks before starting study medication, and the result must be negative. They must also agree to use a medically accepted highly effective contraceptive method during the study period and for 3 months after the last administration of the study drug. For male participants with female partners of childbearing potential, surgical sterilization is required, or they must agree to use an effective contraceptive method during the study period and for 3 months after the last study drug administration;

9) Expected good compliance and willingness to cooperate with the study requirements.

Exclusion criteria:

1) Previous treatment with immune therapy targeting PD-1, PD-L1, CTLA4, or other immune therapies;

2) Previous treatment with TGF-β inhibitors;

3) Known allergy to the study drug or any of its excipients, or a history of severe allergic reactions to other monoclonal antibodies;

4) Treatment received prior to the first study treatment, including:

a) Major surgery within 28 days prior to the first study drug treatment (except for diagnostic biopsies);

b) Use of immunosuppressive drugs within 7 days prior to the first study drug treatment, excluding nasal and inhaled corticosteroids or physiologic doses of systemic corticosteroid hormones (i.e., not exceeding 10 mg/day prednisone or its equivalent);

c) Use of immunomodulatory drugs within 3 weeks prior to the first study drug treatment (e.g., thymosin, interferon, interleukins);
d) Receipt of attenuated live vaccines within 28 days prior to the first study drug treatment or planned vaccination with attenuated live vaccines during the study and within 60 days after the end of study drug treatment;
e) Receipt of antitumor treatment within 28 days prior to the first study drug treatment, except for cases where the interval between the last chemotherapy is at least 2 weeks or five half-lives (whichever is shorter), or the interval between the last targeted therapy is at least five half-lives;

5) Known uncontrolled or symptomatic central nervous system (CNS) metastases;
6) Symptomatic, disseminated, and short-term life-threatening complications risk in advanced patients (including those with uncontrollable large effusions [pleural, pericardial, or abdominal]);
7) Within the past 2 years, a history of other active malignancies, except for the following conditions that have been locally treated and cured: basal cell carcinoma or squamous cell carcinoma of the skin, superficial bladder carcinoma, cervical carcinoma in situ, intraductal breast carcinoma, and papillary thyroid carcinoma;
8) Presence of any active autoimmune disease or a history of autoimmune disease with an anticipated relapse;
9) Known history of allogeneic organ transplantation or allogeneic hematopoietic stem cell transplantation;
10) Human immunodeficiency virus (HIV) infection or known acquired immunodeficiency syndrome (AIDS), untreated active hepatitis (hepatitis B, defined as positive hepatitis B surface antigen [HBsAg] and abnormal liver function with HBV-DNA $\geq$ 500 IU/ml; hepatitis C, defined as positive hepatitis C antibody [HCV-Ab] and detectable HCV-RNA above the limit of detection with abnormal liver function) or concurrent co-infection with hepatitis B and hepatitis C;
11) Occurrence within the past 6 months before entering the study of: myocardial infarction, severe/unstable angina, New York Heart Association (NYHA) class II or higher heart failure, and clinically significant supraventricular or ventricular arrhythmias requiring clinical intervention; poorly controlled hypertension (systolic blood pressure persistently $\geq$150 mmHg or diastolic blood pressure $\geq$100 mmHg);

12) Use of systemic antibiotics for ≥7 days within 4 weeks before the first study drug administration or presence of unexplained fever >38.5°C during the screening period or before the first study drug administration (fever due to tumor causes can be included according to the investigator's judgment);
13) History of gastrointestinal bleeding within the past 6 months or a tendency toward gastrointestinal bleeding, such as esophageal varices, active ulcer lesion, or fecal occult blood ≥(++) (patients with fecal occult blood (+) need to undergo gastroscopy);
14) Urine protein ≥(++) or 24-hour urinary protein > 1.0 g;
15) Inability to swallow the study drug or the presence of factors affecting drug administration and absorption, such as chronic diarrhea (including, but not limited to, irritable bowel syndrome, Crohn's disease, and ulcerative colitis) and intestinal obstruction;
16) Pregnant or lactating women, or female participants of childbearing potential unwilling to use effective contraceptive measures;
17) Known history of neurological or psychiatric disorders, including epilepsy and dementia; known history of substance abuse or drug addiction;
18) Participation in any other drug clinical trial within 4 weeks before the first study drug administration or within 5 half-lives since the last study drug administration;
19) Presence of other severe physical or laboratory abnormalities that may increase the risk of participation in the study or interfere with study results, as determined by the investigator.

Treatment termination criteria

If any of the following events occur, the patient must terminate the study treatment:

1) The subject requests to discontinue the study drug treatment;
2) Poor compliance of the subject, not adhering to the study drug administration as per the protocol;
3) Occurrence of pregnancy in the subject during the course of the study;
4) Disease progression confirmed by the investigator based on the assessment of efficacy;
5) Even with dose adjustments, the subject remains unable to tolerate toxicity or experiences adverse events, laboratory abnormalities, or

	concurrent diseases that, according to the investigator's judgment, make continued participation in the study not in the best interest of the subject;<br>6) Comprehensive deterioration of the subject's health, rendering them unable to continue participating in the trial;<br>7) Significant protocol deviations discovered after enrollment and confirmed by the investigator;<br>8) Other reasons as determined by the investigator that make it impossible to continue the study treatment.
**Study withdrawal criteria**	Reasons for the subject's withdrawal from the study may include:<br>1) Subject's voluntary withdrawal of informed consent and refusal for further follow-up;<br>2) Other circumstances deemed necessary by the investigator for the subject to withdraw from the study;<br>3) Significant protocol design errors discovered during the conduct of the study;<br>4) Lost to follow-up;<br>5) Subject's death;<br>6) Premature termination of the clinical trial by the sponsor or ethics committee;
**Safety evaluation criteria**	Adverse events severity will be assessed according to the CTCAE v5.0 standard. During the trial, an adverse event record form should be accurately filled out, including the time of occurrence, severity, duration, measures taken, and outcome of the adverse events.
**Efficacy assessment**	Subjects will undergo imaging evaluation every 2 cycles (6 weeks) after enrollment. Tumor response assessment will be performed based on RECIST 1.1 criteria. Subjects with a partial response (PR) or complete response (CR) need to undergo efficacy confirmation 4 weeks later.
**Statistical methods**	The main results of this trial will primarily use descriptive statistical methods. For continuous data, mean, standard deviation, median, maximum, minimum values will be presented, while for categorical data, frequency (proportion), rate, and confidence intervals will be provided.

	Safety analysis:<br>Safety analysis will focus on descriptive statistics, analyzing adverse events, serious adverse events, and adverse reactions (where adverse reactions are defined as “definitely related/probably related/indeterminate related to the study drug”) occurring in each dose group. Laboratory test results will be described for cases where abnormalities are observed after treatment, which were normal before the study.<br>Efficacy analysis:<br>Efficacy analysis will estimate the objective response rate (ORR), disease control rate (DCR), and other efficacy endpoints with point estimates and provide 95% confidence intervals representing the overall population using the Kaplan-Meier method. Survival curves will be plotted for median progression-free survival (PFS) and median overall survival (OS). Other secondary efficacy measures such as quality of life will be analyzed descriptively.
**Study duration**	Expected first subject enrollment date: August 2020<br>Expected last subject enrollment date: February 2022<br>Expected study completion date: February 2023
