# Supplementary material for: Clinical and biomarker analyses of SHR-1701 combined with famitinib in patients with previously treated advanced biliary tract cancer or pancreatic ductal adenocarcinoma: a phase II trial
Source: Signal Transduct Target Ther. 2024 Dec 13;9:347. doi: 10.1038/s41392-024-02052-3 (PMC11638339; doi:10.1038/s41392-024-02052-3)
Supplement: Supplementary file 2 — Supplementary Materials [file 41392_2024_2052_MOESM2_ESM.pdf]

Supplementary Materials for

Clinical and biomarker analyses of SHR-1701 combined with famitinib in patients with previously treated advanced biliary tract cancer or pancreatic ductal adenocarcinoma: a phase II trial

Lixia Yi, Haoqi Pan, Zhouyu Ning, Litao Xu, Hena Zhang, Longfei Peng, Yaowu Liu, Yifan Yang, Waimei Si, Ying Wang, Xiaoyan Zhu, Shenglin Huang, Zhiqiang Meng, Jing Xie

Correspondence to: isable624@163.com

This PDF file includes:

Figures. S1 to S4
Tables S1 to S2
Captions for Data S1 to S4

Supplementary Fig. S1

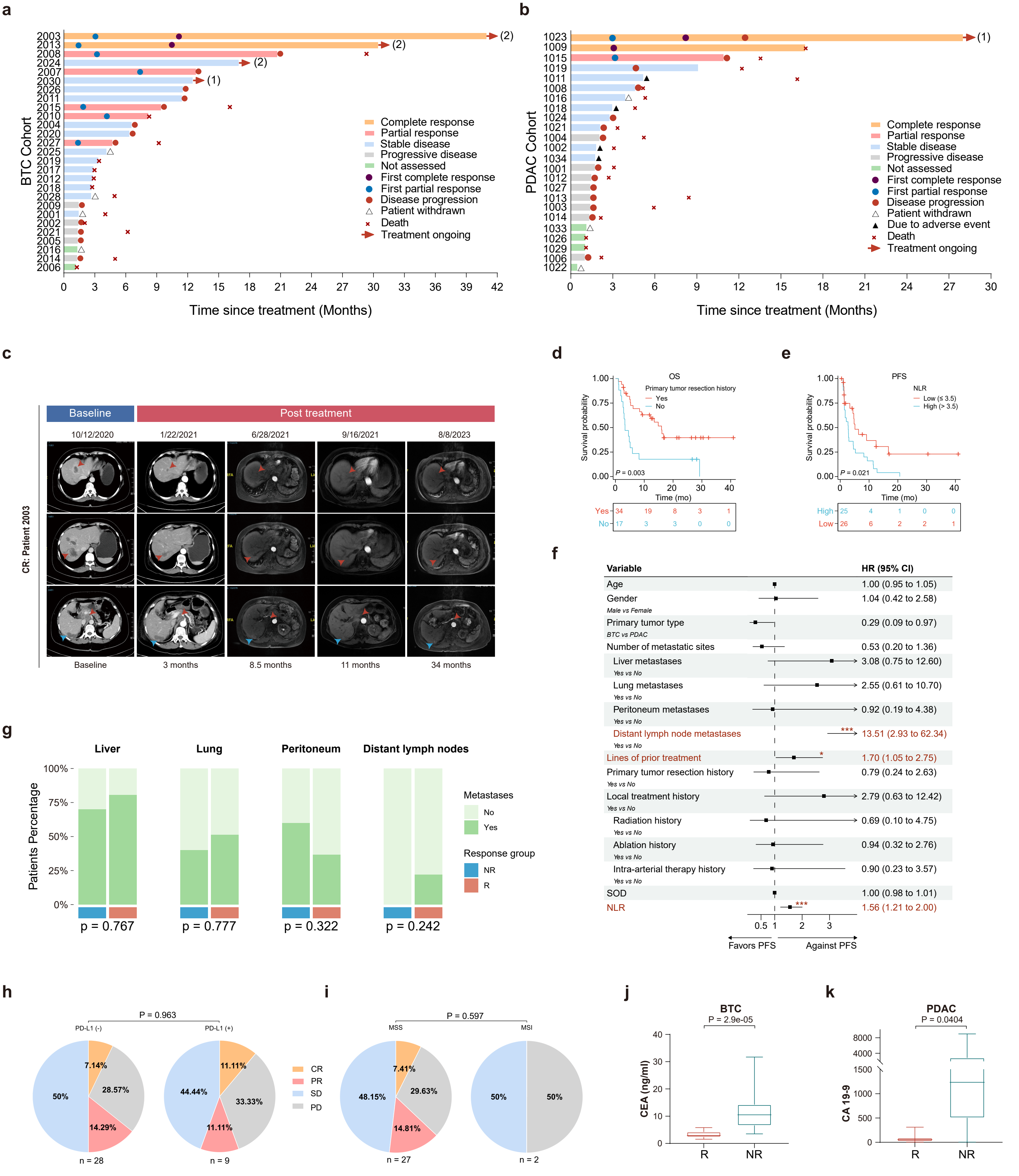

Figure. S1. Clinical outcomes, prognostic factors, and biomarker exploratory analyses of the study.

(a) Swimmer plot of treatment duration, response, and survival outcomes for individual patients in the BTC cohort. (1), patients still receiving active combination treatment of SHR-1701 and famitinib at the data cutoff; (2), patients still receiving famitinib monotherapy at the data cutoff. BTC, biliary tract cancer.

(b) Swimmer plot of treatment duration, response, and survival outcomes for individual patients in the PDAC cohort. Patient 1009 with a DoR of 14 months died due to an accidental fall, unrelated to disease progression. Two patients (no. 1023 and 1019) continued treatment after disease progression. Patient 1023 suspended SHR-1701 due to the COVID-19 pandemic, resulting in tumor recurrence, but resumed combination therapy with stable tumor size. Patient 1019 continued treatment for 6 additional cycles after slow progression until a new brain metastasis appeared. (1), patients still receiving active combination treatment of SHR-1701 and famitinib at the data cutoff. PDAC, pancreatic ductal adenocarcinoma.

(c) Representative magnetic resonance or computed tomography images of the complete response patient 2003 (lesions are marked by triangles; the top row: metastatic lesion 1 in the liver; the middle row: metastatic lesion 2 in the liver; the bottom row: metastatic lesion in the peritoneum [red] and lesion previously treated with microwave ablation in the liver [blue]). Detailed patient background information can be found in Supplementary Table S1.

(d) Kaplan-Meier curve of overall survival (OS) based on history of primary tumor resection in the overall population. Mo, month.

(e) Kaplan-Meier curve of progression-free survival (PFS) based on history of NLR in the overall population. Patients were divided into low NLR (NLR $\leq$ 3.5) and high NLR (NLR > 3.5) groups based on the median NLR value of 3.5. NLR, neutrophil-to-lymphocyte ratio; Mo, month.

(f) Forest plot of hazard ratios (HR) and 95% confidence intervals (CI) for PFS based on various clinicopathological factors. Factors favoring PFS are shown on the right side of the plot, while factors against PFS are shown on the left side. The category of local treatment includes ablation, radiation and intra-arterial therapy. *, $P < 0.05$; **, $P < 0.01$; ***, $P < 0.001$; SOD, sum of diameter; NLR, neutrophil-to-lymphocyte ratio.

(g) Objective response rates stratified by the presence or absence of common metastatic sites in the overall response population. P-values (chi-square test) comparing the response rates between patients with and without metastases at each site are provided. NR, non-responders; R, responders.

(h) Comparison of PD-L1 status in patients. The proportions of CR, PR, SD, and PD were not significantly different between the two groups ($P = 0.963$, Fisher's exact test). CR, complete response; PR, partial response; SD, stable disease; PD, progressive disease.

(i) Comparison of microsatellite stability (MSS) status in patients. The proportions of CR, PR, SD, and PD were not significantly different between the two groups ($P = 0.597$, Chi-square test).

(j) Comparison of carcinoembryonic antigen (CEA) levels in responders (R) and non-responders (NR) of BTC patients ($P = 2.9e-05$, Mann-Whitney U test).

(k) Comparison of carbohydrate antigen 19-9 (CA 19-9) levels in responders (R) and non-responders (NR) of PDAC patients ($P = 0.0404$, Mann-Whitney U test).

Supplementary Fig. S2

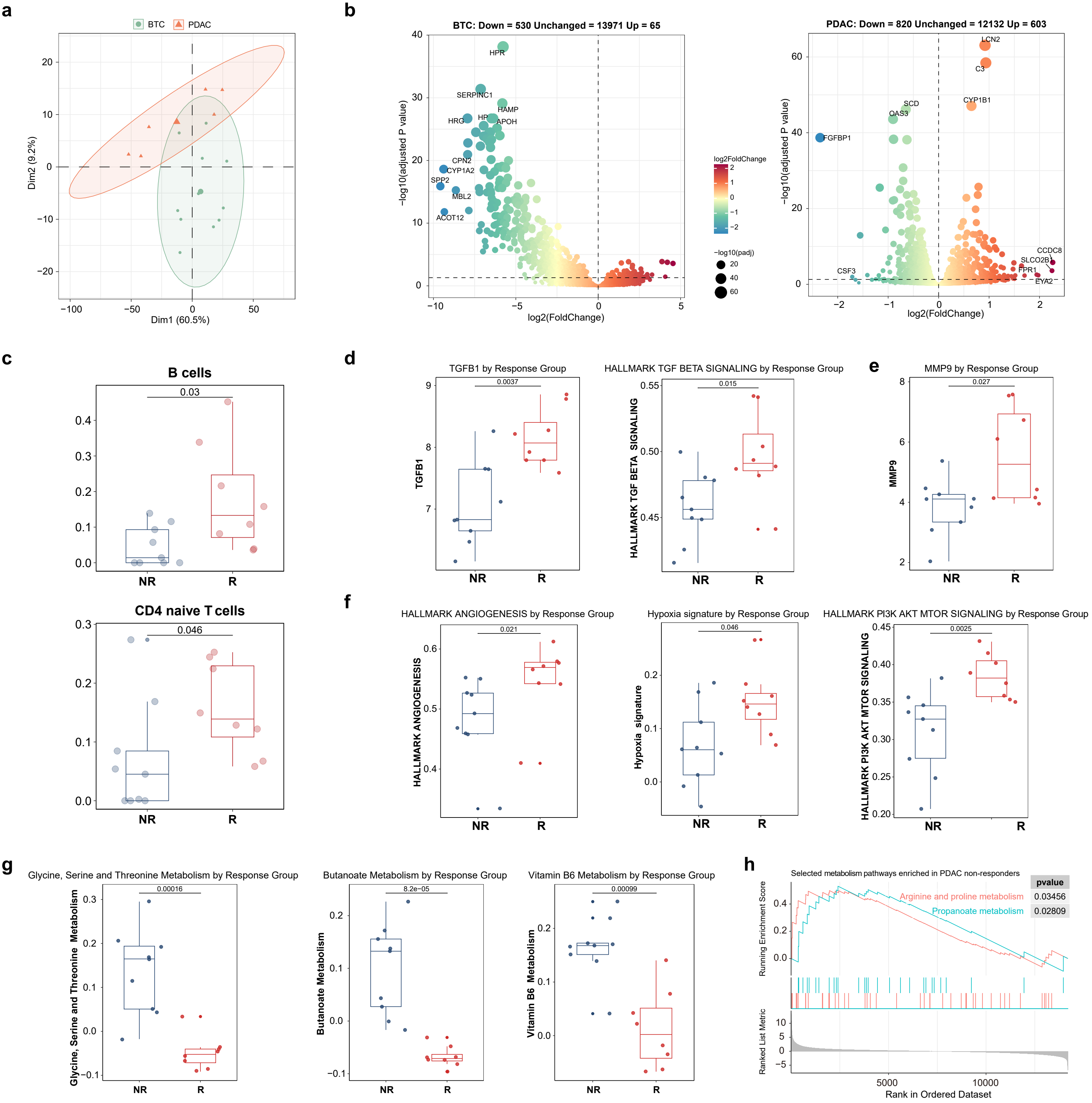

Figure. S2. Transcriptomic analysis of baseline primary tumor samples.

(a) Principal component analysis (PCA) plot of RNA-seq data between BTC and PDAC patients.
(b) Volcano plots displaying differentially expressed genes between responders and non-responders in the BTC (left) and PDAC (right) cohorts. Genes with a fold change > 1 and adjusted P-value < 0.05 are highlighted in red (upregulated in responders) and blue (downregulated in responders).
(c) Comparison of the estimated abundance of B cells and $CD4^+$ naive T cells in the tumor microenvironment (TME) between responders (R) and non-responders (NR) using xCell deconvolution analysis. Responders showed a trend towards higher proportions of B cells (P = 0.03, Mann-Whitney U test) and $CD4^+$ naive T cells (P = 0.046, Mann-Whitney U test) compared to non-responders.
(d-f) Comparison of immunosuppressive gene expression and signaling pathway levels between responders (R) and non-responders (NR) in the combined BTC and PDAC cohorts (Mann-Whitney U test).
(g) Comparison of certain metabolic pathway levels between responders (R) and non-responders (NR) in the combined BTC and PDAC cohorts (Mann-Whitney U test).
(h) Gene set enrichment analysis of arginine and propanoate metabolism KEGG pathways in non-responders with pancreatic ductal adenocarcinoma (PDAC).

Supplementary Fig. S3

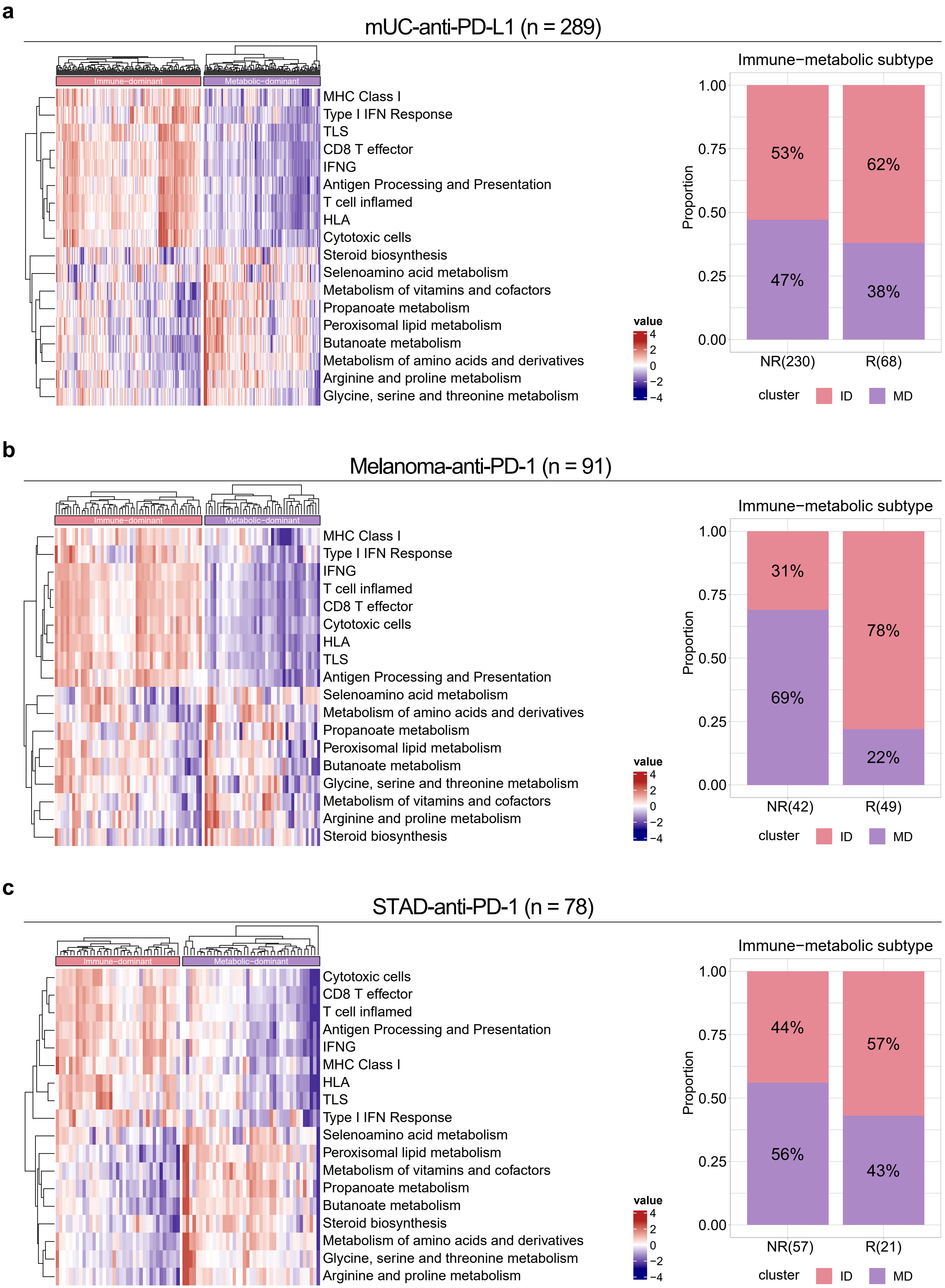

Figure. S3. **Immune-metabolic stratification in external validation cohorts.**

(a) Left: Heatmap of ssGSEA scores for immune signatures and metabolic pathways in the mUC-anti-PD-L1 cohort (n = 289). Patients were stratified into immune-dominant (ID, left) and metabolic-dominant (MD, right) subgroups. Right: Proportion of patients in the immune-dominant (ID) and metabolic-dominant (MD) subgroups within the non-responder (NR) and responder (R) groups. mUC, metastatic urothelial carcinoma.
(b) Left: Heatmap of ssGSEA scores for immune signatures and metabolic pathways in the Melanoma-anti-PD-1 cohort (n = 91). Right: Proportion of patients in the ID and MD subgroups within the NR and R groups.
(c) Left: Heatmap of ssGSEA scores for immune signatures and metabolic pathways in the STAD-anti-PD-1 cohort (n = 78). Right: Proportion of patients in the ID and MD subgroups within the NR and R groups. STAD, stomach adenocarcinoma.

Supplementary Fig. S4

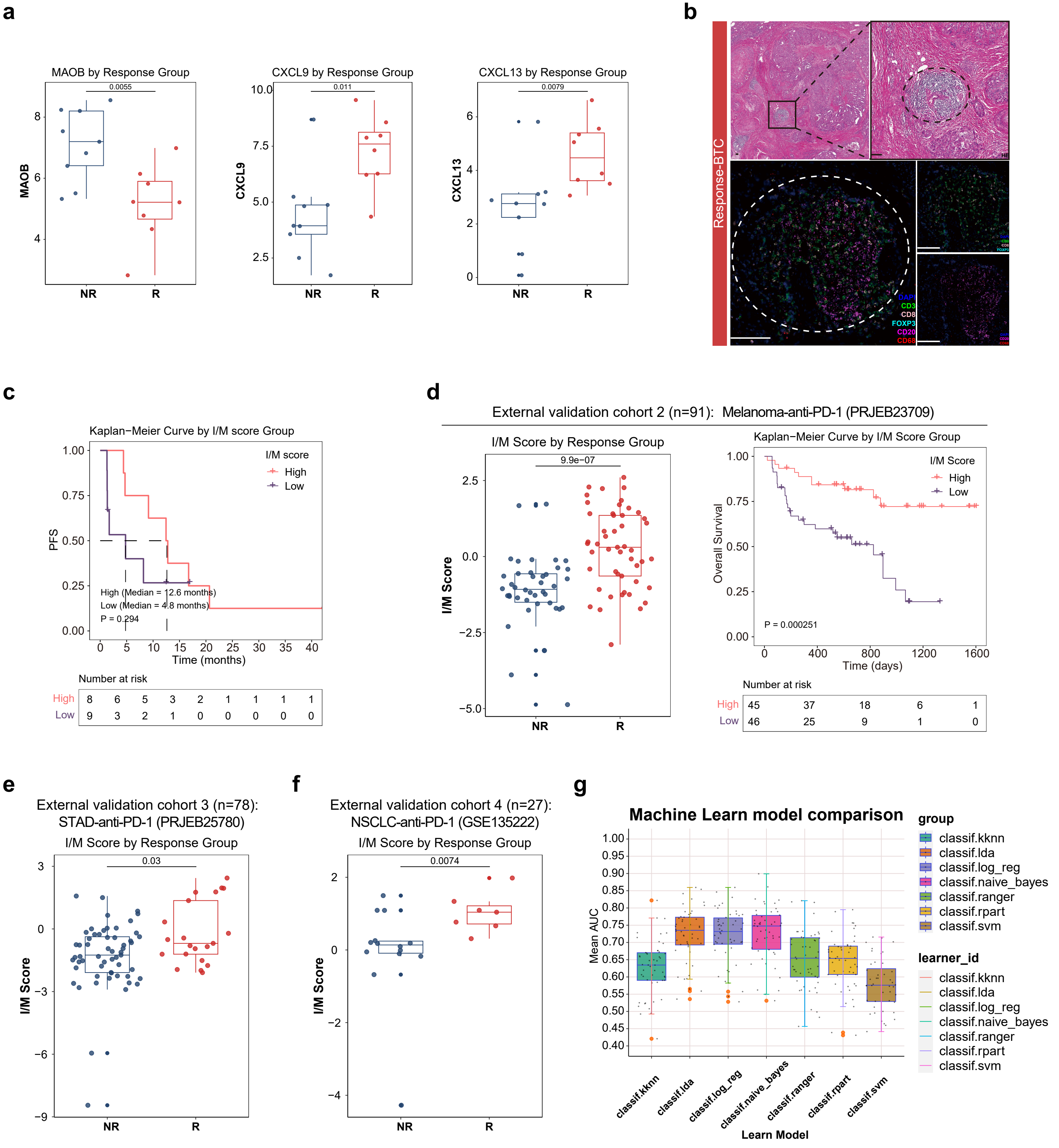

Figure. S4. Immune/metabolism (I/M) score and its predictive performance in various cancer types.

(a) Expression levels of the represented genes used to calculate the I/M score between responders (R) and non-responders (NR) from our cohort (Mann-Whitney U test).
(b) Representative mIHC images of tumor samples from a BTC responder. Color-coded for immune cell populations was as illustrated, with the TLS region circled by a dashed line. The scale bar at the bottom left corner of the mIHC images represents 0.1 mm. TLS, tertiary lymphoid structure.
(c) Kaplan-Meier curves for progression-free survival (PFS) stratified by I/M score group (high vs. low), with median PFS and P-value (0.294, log-rank test). Number at risk table was included.
(d) I/M scores in responders (R) vs. non-responders (NR) (left) and Kaplan-Meier curves for overall survival (OS) stratified by I/M score group (high vs. low) (right) in the Melanoma-anti-PD-1 external validation cohort (n=91). The plots included P-values (Mann-Whitney U test for I/M scores; log-rank test for OS) and a number at risk table.
(e) I/M scores in responders (R) vs. non-responders (NR) in the STAD-anti-PD-1 external validation cohort (n=78; Mann-Whitney U test). STAD, stomach adenocarcinoma.
(f) I/M scores in responders (R) vs. non-responders (NR) in the NSCLC-anti-PD-1 external validation cohort (n=27; Mann-Whitney U test). NSCLC, non-small cell lung cancer.
(g) Comparison of various machine learning models used for developing the prediction model based on the I/M score. The bar plot showed the mean AUC values for each model. The naive bayes model achieved the highest mean AUC. AUC, area under the curve; KNN, k-nearest neighbors; LDA, linear discriminant analysis; RPART, recursive partitioning and regression trees; SVM, support vector machine.

Table S1. Treatment-related adverse events in all treated patients.

	Total (n=51)	
	Any grade	Grade 3-4
**TRAEs**	45 (88.2)	15 (29.4)
**TRAEs occurring in ≥ 5 % of patients**		
**Renal and urinary**		
Proteinuria	24 (47.1)	1 (2.0)
Urinary occult blood positive	16 (31.4)	0
Blood creatinine increased	4 (7.8)	0
**Hematologic**		
Anemia	20 (39.2)	7 (13.7)
Thrombocytopenia	13 (25.5)	1 (2.0)
**Gastrointestinal**		
Diarrhea	15 (29.4)	1 (2.0)
Occult blood positive	7 (13.7)	0
Gingival bleeding	7 (13.7)	0
Mouth haemorrhage	6 (11.8)	0
Mouth ulceration	4 (7.8)	0
Vomiting	4 (7.8)	0
Nausea	3 (5.9)	0
**Hepatobiliary function**		
Aspartate aminotransferase increased	15 (29.4)	1 (2.0)
Alanine aminotransferase increased	13 (25.5)	2 (3.9)
Blood bilirubin increased	7 (13.7)	0
Bilirubin conjugated increased	3 (5.9)	2 (3.9)
**Skin**		
Rash	14 (27.5)	2 (3.9)
Palmar-plantar erythrodysesthesia syndrome	6 (11.8)	0
**Vascular**		
Hypertension	13 (25.5)	4 (7.8)
Epistaxis	7 (13.7)	0
**Metabolism**		
Hypoalbuminemia	12 (23.5)	0
Decreased appetite	9 (17.6)	0
Blood uric acid increased	3 (5.9)	0
**Endocrine**		
Hypothyroidism	8 (15.7)	0
**General disorders**		
Asthenia	4 (7.8)	0
**Other**		
Troponin increased	4 (7.8)	0
N-terminal prohormone brain natriuretic peptide increased	3 (5.9)	0

TRAEs, treatment-related adverse events.

Table S2. Potential Immune-related adverse events.

	**Total (n=51)**	
	Any grade	Grade 3-4
**Potential irAEs**	16(31.4)	2(3.9)
Rash	12(23.5)	2(3.9)
Hypothyroidism	7(13.7)	0
Alanine aminotransferase increased	1(2.0)	0
Aspartate aminotransferase increased	1(2.0)	0
Myocarditis	1(2.0)	0

irAEs, immune-related adverse events.

Data S1. (separate file)

Baseline characterization and response details of patients achieving complete response (CR) in the study.

Data S2. (separate file)

GO pathway enrichment analysis of differentially expressed genes between responders and non-responders.

Data S3. (separate file)

Gene set signatures related to immune and metabolism.

Data S4. (separate file)

GSEA results of the pathways from KEGG and REACTOME.
